# How Do Patients Want Us to Use the Computer During Medical Encounters?—A Discrete Choice Experiment Study

**DOI:** 10.1007/s11606-021-06753-1

**Published:** 2021-04-26

**Authors:** Cédric Lanier, Melissa Dominicé Dao, Dave Baer, Dagmar M. Haller, Johanna Sommer, Noëlle Junod Perron

**Affiliations:** 1 Primary Care unit (UIGP), BFM local 4091, Centre Médical Universitaire de Genève, University of Geneva, Geneva, Switzerland; 2 Department of Community Medicine, Primary Care and Emergency Medicine, Geneva University Hospitals, Geneva, Switzerland; 3 Cité Générations, Onex, Switzerland; 4 Unit of Development and Research in Medical Education (UDREM), University of Geneva, Geneva, Switzerland

**Keywords:** Computer use, Electronic health record, Primary care, Communication skills, Patient-physician relation

## Abstract

**Background:**

Primary care physicians (PCPs) now widely use electronic health records (EHRs) during medical encounters. Experts in clinical communication issued recommendations for a patient-centered use of EHRs. However, they have never been validated by patients themselves.

**Objective:**

To explore patients’ preferences regarding physicians’ EHR-related behaviors.

**Design:**

Discrete choice experiment study.

**Patients:**

French-speaking patients waiting for a medical consultation at two outpatient clinics in Geneva, Switzerland.

**Main Measures:**

We invited patients to watch videos displaying 2 or 3 variations of four specific EHR-related behaviors and asked them to indicate which one they preferred. EHR-related behaviors were (1) typing: continuous/intermittent/handwriting in biomedical or psychosocial focused consultations; (2) maintaining contact while typing: visual/verbal/both; (3) signposting the use of EHR: with/without; (4) position of physicians’ hands and bust: on the keyboard and towards the patient/away from the keyboard and towards the patient/on the keyboard and towards the screen.

**Key Results:**

Three hundred thirty-six patients participated (response rate 61.4%). They preferred intermittent typing versus handwriting or continuous typing for biomedical issues (32.7%; 95% CI: 26.0–40.2% vs 31.6%; 95% CI: 24.9–39.0% or 14.9%; 95% CI: 10.2–21.1%) and psychosocial issues (38.7%; 95% CI: 31.6–46.3% vs 24.4% 95% CI: 18.4–31.5% or 17.9%; 95% CI; 12.7–24.4%). They favored visual and verbal contact (38.9%; 95% CI: 31.9–46.3%) over verbal (30.3%; 95% CI: 23.9–37.5%) or visual contact only (11.4%; 95% CI: 7.5–17.1%) while the doctor was typing. A majority preferred signposting the use of EHR versus no signposting (58.9%; 95% CI: 53.5–64.0% vs 34.8%; 95% CI: 29.9–40.1%). Finally, half of the patients (49.7%; 95% CI: 42.0–57.4%) favored the position with the physician’s bust towards the patient and hands away from the keyboard.

**Conclusions:**

Our study shows that patients’ preferences regarding EHR-related behaviors are in line with most experts’ recommendations. Such recommendations should be more consistently integrated into under- and postgraduate communication skills training.

**Supplementary Information:**

The online version contains supplementary material available at 10.1007/s11606-021-06753-1.

## Introduction

In the past 25 years, health professionals have increasingly become accustomed to the use of electronic health records (EHRs) during clinical encounters. EHR use improves the quality of biomedical data gathering. It facilitates medical information sharing with patients and between the different health providers, and reduces medical errors.^1–5^

Patients generally consider EHR as useful and important and are satisfied with their use .^3,6–8^ However, the use of EHR during clinical encounters tends to influence physicians’ and patients’ behaviors and communication.^9^ Some physicians may spend their time using the EHR instead of establishing the consultation’s agenda at the beginning of the encounter.^10^ The EHR use increases moments of silence and decreases physician-patient visual interaction.^3,11–13^ Physicians spend a quarter of the encounter time gazing at the screen and this time appears to be inversely correlated with their interest in patients’ psychosocial and emotional discourse.^12–14^ Furthermore, depending on their visual access to the screen, patients do not know what their physician is doing and dislike this situation.^13^ Some authors observed that EHR use tends to amplify physicians’ good or poor communication skills.^12,15,16^

Other factors appear to influence physicians’ EHR use and patient-physician relationship. They include physicians’ typing and computer skills, clinical experience, personal style of EHR use (particularly verbal and non-verbal behaviors), spatial arrangement of both the computer and the screen, and finally the design of the EHR.^17–21^

Based on these observations, authors and experts in medical communication issued recommendations in order to facilitate patient-physician communication while using EHR during the encounter (see Box 1) ^12,22,23^. These recommendations, based on principles of patient-centered approach, take into account physicians’ verbal and non-verbal communication skills described in most physician-patient communication frames of reference ^24,25^. They are summarized in Text Box 1.

Text Box 1. Summary of recommendations on how to use the electronic health record (EHR). Adapted from^22,23^

**Table:** 

• Open the EHR before inviting the next patient in the consultation room	
• Explore and negotiate the patient’s agenda before using the EHR	
• Move the computer’s screen to offer the patient a visual access to the screen/EHR during the clinical encounter (when possible)	
• Keep a position facing the patient most of the time with hands away from the keyboard	
• Indicate when the EHR is used and explain what is done with the EHR = signposting the use of EHR	
• Indicate to the patient where the physician’s attention is directed (EHR or the patient) by using verbal and non-verbal clues	
• Invite the patient to consult the information displayed on the screen	
• Give full attention to the patient by not using the EHR when he/she expresses emotions or psychosocial issues	
• Type during appropriate time sets (i.e., just before or after the physical examination)	

Medical associations and experts recommend integrating these EHR-related communication skills into medical training.^26–28^ Despite the apparent effectiveness of such training, medical students and physicians remain insufficiently trained in how to use EHR in a patient-centered way .^29–32^

To date, studies have mostly surveyed patients’ satisfaction regarding physicians’ ways of using the computer. They have also explored the functions and roles physicians attribute to the computer .^33–35^ To our knowledge, no studies have specifically explored whether patients agree with experts’ recommendations regarding EHR use. In particular, none has explored patients’ perspectives about specific EHR-related behaviors such as physicians’ position and attitude when typing or using the EHR .^36,37^

The aim of our study was thus to explore patients’ preferences regarding physicians’ EHR-related behaviors, and to assess the extent to which these preferences mirror experts’ recommendations regarding EHR use.

## Methods

### Design and Setting

We conducted a discrete choice experiment^38^ between April and July 2018 in Geneva, Switzerland. The discrete choice experiment is a quantitative survey-based approach commonly used in health care to elicit patients’ preferences regarding health care .^39,40^ Patients are presented with different options (or, as in our case, scenarios) which differ only according to specific attributes, and asked to state their preference in relation to these options. Their choice reflects the value patients place on the specific attributes that are being tested in the experiment.

Patients were recruited from two outpatient settings: (1) a walk-in clinic at Geneva University Hospitals which provides 35,000 consultations a year for patients presenting with medical and traumatic problems that do not require hospitalization—it is a training center for 40 residents training in general internal medicine; (2) a walk-in clinic situated in a suburb of Geneva, which provide 36,000 consultations a year for patients presenting with minor medical and traumatic problems—it is also a training center for 8 residents training in general internal medicine.

### Participants and Procedure

Following informed consent, French-speaking patients, aged > 18 years old, waiting for a medical consultation, were invited by research assistants to participate in an online survey on a tablet provided by the research assistant. They were informed they would have to watch videos displaying variations of practitioner’s EHR-related behaviors. They were then asked to indicate which one they preferred. Exclusion criteria were not being able to read and understand French.

Ten research assistants were involved in the patient’s recruitment between April and July 2018. They were recruited among 2nd and 3rd year medical students and were trained on how to approach patients, explain the goals of the study, obtain informed consent, use the tablet, and provide help if necessary during an individual session. They included ca 33 patients each (median 33; IQR 19).

### Development of Videos Displaying EHR-Related Behaviors

Based on recommendations for EHR use ,^22,23^ we identified 4 distinct patterns of EHR use (including one with two variations depending on the content of the clinical encounter) regarding typing, contact maintenance, signposting (indicate when and why the EHR is used), and physician’s position (see Table 1). Each EHR-related behavior pattern was related to a common primary care complaint.

**Table 1:** Distribution of EHR-Related Behaviors, Clinical Scenario, and Physicians’ and Simulated Patients’ Characteristics and EHR-Related Behaviors

Video sequence (mean duration of video [s])	EHR-related behaviors while typing			Clinical scenario	Physician	Patient
A1 74	Continuous typing	Intermittent typing (while summarizing)	Handwriting	Back pain (biomedical content)	53-year-old woman	70-year-old man
A2 96	Continuous typing	Intermittent typing *(while summarizing)*	Handwriting	Hand injury (psychosocial content)	35-year-old man	30-year-old woman
B 79	Visual contact	Verbal contact	Visual + verbal contact	Headache	47-year-old woman	45-year-old woman
C 70	Signposting the EHR use	--	No signposting of the EHR use	Cutaneous eruption and immunization	55-year-old man	18-year-old man
D 88	Bust towards the patient and hands on the keyboard	Bust towards the computer and hands on the keyboard	Bust towards the patient and hands away from the keyboard	Dyspepsia	46-year-old woman	80-year-old woman

*EHR*, electronic health record

The physicians acting in the videos were five clinical teachers involved in communication skills’ training in undergraduate curriculum at the Geneva Faculty of Medicine. They were of different ages (35, 46, 47, 53, and 55 years) and gender (3 women and 2 men). They were expected to demonstrate good verbal communication skills independently of the use of EHR. They were matched with simulated patients of different ages and gender working at the Geneva Faculty of Medicine, with the aim of limiting gender or age biases. The distribution of complaints, physicians, and patients is shown in Table 1.

We wrote a script for every EHR-related behavior and their tested variations portrayed in the videos. For each EHR-related behavior, two or three sequences were recorded, with each sequence showing a variation of this behavior. Clinical teachers were asked to closely follow the script and replicate exactly the same verbal and non-verbal communication for each of the 2-3 sequences, except for the desired EHR-related behavior variations (i.e., body direction and hand positions …). The simulated encounters were videotaped from a patient perspective by a professional videographer in the presence of two investigators in order to ensure that the acting physicians respected the instructions. The sequences were repeated until the verbal and non-verbal communication displayed matched the research goals (same verbal and non-verbal communication unrelated to EHR-related behaviors and variations in EHR-related behaviors). In order to check such replicability, we asked experienced primary care physicians, blinded to the study objectives, to identify and validate the videotaped variations of the different EHR-related behaviors. We also asked them to check whether acting physicians displayed similar verbal and non-verbal communication unrelated to the EHR in the different sequences for each scenario.

### Procedure

Participants were asked to watch three different videos integrated into an online survey on a tablet in the waiting room .^41^ They were asked to watch the videos as if they were the patient in the consultation. It took them approximatively 15 min to complete the survey: 10 min to watch videos and give their preferences and 5 min to answer socio-demographic information as well as data in relation to their own and their physician’s computer use. Each set of videos included two (for signposting) or three different sequences displaying variations of the tested EHR-related behavior. The sets were organized in the following way: the first 80 patients watched the video set displaying sequences A1-B-C; the next 80 patients watched the set including videos C-D-A2, the following 80 watched the set with videos A1-D-C, and finally 80 patients watched a set containing video sequences B-C-A2 (see Table 1). In each video set, the different sequences were presented in a random order to minimize a possible bias in relation to the position of a sequence within the set. After watching the video set, the patients selected their preference directly on the tablet (see Discrete choice experiment (DCE) online survey in Appendix 1).

### Outcomes, and Other Measures

The main outcome was patients’ preference among two or three variations regarding the four EHR-related behaviors. However, if they could not reach a decision, they could indicate two preferences. At the end of the survey, patients were also asked to provide socio-demographic information as well as data in relation to their own and their physician’s computer use (see Table 2) (Appendix 2). These variables were chosen because of their potential impact on EHR use preference.^21,42,43^ We also recorded study location (hospital versus community emergency service) in order to account for potential location-related biases.

**Table 2:** Participants’ Socio-Demographic and Experiences of Computer Use

	*n* (%)
Participants • Response rate	336 (100) (61.4)
Gender	
• Female	185 (55.1)
Age	
• <30	123 (36.6)
• 30–49	111 (33.0)
• >50	102 (30.4)
1st language	
• French	256 (76.2)
• Other	80 (23.8)
Nationality	
• Swiss	184 (54.8)
Level of education	
• Obligatory	68 (20.2)
• Secondary	141 (42.0)
• Tertiary	127 (37.8)
Frequency of consultations/year	
• 0-4	281 (83.6)
• ≥5	55 (16.4)
Computer use	
• < 1 per week	70 (20.8)
• ≥ 1 per week	266 (79.2)
Own physician’s use of computer	
• Yes	226 (67.3)
Favorable of physician’s use of computer	
• Yes	267 (79.5)
Familiar with computers, tablets, or smartphone	
• Yes	268 (79.8)

### Sample Size Estimate

We originally estimated our sample size for this study in order to have a 90% power of detecting a 20% difference in the proportion of patients choosing a specific sequence compared to the null hypothesis (50% of participants choose the sequence). This led to a sample size of 168 patients. Given that each patient had to assess only three of the five different video sets, we doubled this number to include 330 patients. This estimate turned out to be well above the “rule of thumb” proposed in the literature about discrete choice experiments.^38^

### Statistical Analysis

We used proportions to summarize patients’ preferences for a sequence in each video set. We then used multinomial logistic regression to analyze predictors of patients’ choices of a sequence (and therefore of a specific EHR-related behavior) within each video. Multinomial logistic regression provides the possibility to measure associations of independent variables with a categorically distributed outcome, i.e., in our case, three different levels of EHR-related behaviors (two in video C). The different levels of the outcome are compared to a baseline condition, which can be chosen arbitrarily. We chose the most commonly used behavior before the introduction of EHRs as a baseline condition. No typing (i.e., handwriting) was chosen as a baseline condition to be compared with intermittent or continuous typing, no signposting was compared to signposting, bust towards the patient and hands away from the keyboard was the baseline condition against which the other two positions were compared, and visual contact was chosen as a baseline condition compared to verbal only or visual and verbal contact. All the patient socio-demographic and computer-related variables listed in Table 2 were introduced as independent variables into the model.

## Results

Three hundred thirty-six patients participated and each watched three different video sets. As shown in Table 2, they were mostly female and younger than 50 years old. The majority were native French speakers and more than half were Swiss nationals. The majority of the patients had completed secondary or tertiary education. Most of the patients had consulted their primary care physician (PCP) less than 4 times during the previous 12 months. The majority of the patients had a PCP who used a computer during the encounter and most were in favor of its use. Seventy-nine percent of the patients used a computer more than once per week and were familiar with other electronic devices.

### Patients’ Preferences Regarding Doctors’ EHR-Related Behaviors

A majority of the patients preferred intermittent typing to handwriting, and overall to continuous typing (Table 3). However, the tendency was less marked with biomedically focused than psychosocially focused clinical encounters. Patients favored regular visual and verbal contact and verbal contact over visual contact alone while typing, as well as signposting the use of EHR as compared to no signposting. Finally, a higher proportion of the patients chose the position with the physician’s bust towards the patient and hands away from the keyboard compared to the two other positions.

Patients could also indicate a double choice in case of hesitation: the number of patients choosing two options was low for signposting (6.3%) and averaged 20% for the other EHR-related behaviors. The distribution of double choices and the relative risk ratios for these choices is shown in Table 3.

**Table 3:** Patients’ Preferences Regarding EHR-Related Behaviors

Behavior and variations	Patient’s preferences	
	*n* (%; 95% CI)	RRR (*p* value)*
Typing - Biomedical content (video A1)		
• Handwriting	53 (31.6; 95% CI: 24.9–39.0)	Baseline
• Intermittent typing	55 (32.7; 95% CI: 26.0–40.2)	1.04 (0.85)
• Continuous typing	25 (14.9; 95% CI: 10.2–21.1)	0.47 (0.002)
• Double choice	35 (20.8; 95% CI: 15.3–27.7)	0.66 (0.06)
Handwriting or intermittent typing	*14 (8.3; 95% CI: 5.0–13.6)*	
Handwriting or continuous typing	*10 (5.9; 95% CI: 3.2–10.7)*	
Intermittent or continuous typing	*11 (6.5; 95% CI: 3.6–11.5)*	
Typing - Psychosocial content (video A2)		
• Handwriting	41 (24.4; 95% CI: 18.4–31.5)	Baseline
• Intermittent typing	65 (38.7; 95% CI: 31.6–46.3)	1.58 (0.02)
• Continuous typing	30 (17.9; 95% CI: 12.7–24.4)	0.73 (0.19)
• Double choice	32 (19.0; 95% CI: 13.7–25.8)	0.78 (0.29)
Handwriting or intermittent typing	*12 (7.1; 95% CI: 4.1–12.2)*	
Handwriting or continuous typing	*9 (5.4; 95% CI: 2.8–10.0)*	
Intermittent or continuous typing	*11 (6.5; 95% CI: 3.6–11.5)*	
Contact (video B)		
• Visual contact only	20 (11.4; 95% CI:7.5–17.1)	Baseline
• Verbal contact only	53 (30.3; 95% CI: 23.9–37.5)	2.65 (<0.001)
• Visual + verbal contact	68 (38.9; 95% CI: 31.9–46.3)	3.4 (<0.001)
• Double choice	34 (19.4; 95% CI: 14.2–26.0)	1.7 (0.06)
Visual + verbal or verbal contact only	*17 (9.7; 95% CI: 6.1–15.1)*	
Visual contact only or verbal contact only	*10 (5.7; 95% CI: 3.1–10.3)*	
Visual + verbal or visual contact only	*7 (4.0; 95% CI: 1.9–8.2)*	
Signposting (video C)		
• No signposting	117 (34.8; 95% CI: 29.9–40.1)	Baseline
• Signposting	198 (58.9; 95% CI: 53.5–64.0)	1.69 (<0.001)
• Double choice	21 (6.3; 95% CI: 4.1–9.4)	0.18 (<0.001)
Body position (video D)		
• Bust facing the patient and hands off the keyboard	80 (49.7; 95% CI: 42.0–57.4)	Baseline
• Bust facing the patient and hands on the keyboard	32 (19.9; 95% CI: 14.4–26.8)	0.4 (<0.001)
• Bust facing the computer and hands on the keyboard	21 (13.0; 95% CI: 8.6–19.2)	0.26 (<0.001)
• Double choice	28 (17.4; 95% CI: 12.2–24.1)	0.35 (<0.001)
Bust facing the patient and hands off the keyboard or bust facing the computer and hands on the keyboard	12 (7.4; 95% CI:4.3–12.7)	
Bust facing the patient and hands on the keyboard or bust facing the computer and hands on the keyboard	12 (7.4; 95% CI:4.3–12.7)	
Bust facing the patient and hands off the keyboard or bust facing the patient and hands on the keyboard	4 (2.5; 95% CI: 0.9–6.5)	

*CI*, confidence interval; *using multinomial logistic regression

### Factors Associated with Patients’ Choices Regarding EHR-Related Behaviors

The multivariate analyses showed that among all the patients’ characteristics, only a positive attitude towards EHR was consistently associated with a preference towards intermittent or continuous typing (Supplementary Table). Other patients’ characteristics such as listed in Table 2 were not associated with any specific preference in relation to physicians’ EHR-related behaviors. In addition, the order of display of the video sequences, the research assistant, and the location in which the study took place (university versus community walk-in clinic) were not associated with patients’ preferences, except for the “visual/verbal contact” video set. A lower relative risk of choosing “visual + verbal contact” or “double choice” was observed at the walk-in clinic of Geneva University Hospitals.

## Discussion

The aim of our study was to explore patients’ preferences regarding physicians’ EHR-related behaviors. Most patients involved in the study were young or middle-aged and in good health condition. They were mostly familiar with the use of computers and electronic devices and were in favor of the physician’s EHR use during medical encounters. A majority of the patients chose the most patient-centered EHR-related behaviors such as physicians’ visual and verbal contact while typing, signposting the EHR use, and facing the patient with hands away from the keyboard. Interestingly, intermittent typing on keyboard was also preferred to handwriting. The only factor significantly associated with a preference towards active use of the computer in the consultation was a positive attitude towards EHR use.

### Intermittent Typing Shows Patients That Their Physician Is Paying Attention

Patients preferred intermittent typing on the keyboard to continuous typing or handwriting. It confirms that computers can be used in a patient-centered manner.^15,22,23,32,33,44^ Intermittent typing may even enhance the therapeutic relationship for several reasons: intermittent typing (mainly during summarizing) allows patients to notice when physicians direct their attention to the EHR or to the patient.^22,36^ Explicitly documenting patients’ information while summarizing may even reinforce patients’ perceptions that the physician is giving importance to their words. The fact that patients preferred intermittent typing for the psychosocially focused encounter suggests that using EHR when discussing emotional and psychosocial issues is possible and may be considered to be patient-centered as long as pauses in typing are made to preserve contact with patient. These findings are new since previous studies reported inversely negative relationships between EHR use and physicians’ interest for patient’s psychosocial and emotional discourse.^12–14^

### Visual and Verbal Contact Maintains the Fluency of the Conversation

Patients preferred visual and verbal contact to verbal or visual contact alone while typing. Visual and verbal contact is the most natural and fluent way of staying in contact during a conversation. Visual contact itself during a dialogue is known to activate “social” neural pathways related to the intention to communicate during a face-to-face dialogue .^45,46^ As mentioned by medical communication experts, a visual contact allows maintaining close contact while using EHR .^22,35,36^ Moments of silence without visual or verbal contact exceeding 5 s can cause a break in the patient’s discourse and a change of topic .^3,32,47^ Previous studies have reported poorer quality of patient-physician communication and a decrease of patients’ involvement when physicians were silent while using the computer .^35,36^ A decrease in visual contact while using EHR does not seem to be necessarily related with patients dissatisfaction ^48^ and it is not clear whether visual contact has a superior “value” over verbal contact during physician-patient interaction .^13,32,49,50^ In our study, almost a third of patients preferred verbal contact alone while using EHR. Talking to patients while entering or extracting data seems to compensate for the decrease in visual contact ^35^, especially if physicians summarize patients’ information or explain what they are doing while typing.

### A Good Spatial Organization Is Needed to Face the Patient Most of the Time

Unsurprisingly, patients favored the “bust towards the patient and hands away from the keyboard” position as it is the most patient-centered position. It means that a good spatial organization is needed to face the patient most of the time and favor good non-verbal communication.^24,34,37,51,52^

### Signposting Gives Direction and Structure to the Consultation

Finally, our results confirm the importance of signposting while using the EHR in order to inform the patient about the aim and the structure of the consultation .^22,32,33,43,53,54^ As mentioned earlier, it also compensates for reduced visual contact and permits to keep a fluent conversation ^35^.

Finally, the multivariate analyses showed that, among all patients’ characteristics, only a positive attitude towards EHR was significantly associated with a preference towards using the keyboard during the consultation. These results echo other studies showing that patients are satisfied with physicians’ use of EHR whatever their personal characteristics .^6,8,55,56^

In summary, our results confirm that patients are sensitive to variations of physicians’ EHR-related behaviors and prefer patient-centered EHR uses as recommended by communication experts. Our study suggests that using the computer is not an obstacle to patient-centeredness, if used appropriately and if other elements of good communication are present.^44^ To our knowledge, this is the first study in which real patients validate recommendations regarding typing style and body position while using the EHR.^36^

### Limitations

Our study has several limitations. It is possible that physicians who played in the videotaped clinical encounters unconsciously used different non-verbal behaviors in the two or three sequences displaying different styles of EHR use. However, this potential bias seems to be limited since the external reviewers who were asked to validate the content of our videos did not identify variations in physician-patient communication quality beyond the modelled EHR-related behavior.

We wrote the clinical encounter scenarios to reflect the situation of a first encounter between a patient and a physician. Thus, our findings do not necessarily extend to the encounters within the continuity of care.

Admittedly, the use of video-based DCE does not seem common, and we cannot be certain that the choices made by patients would have been similar if exposed to real-life consultations. An alternative would have been to ask patients to make a choice between different written vignettes, thus making the tested attributes more explicit than in our videos. This would, however, have led the findings to be more dependent on patients’ understanding of the written descriptions. Written vignettes seemed less realistic to us, and would have excluded patients with reading difficulties.

We used clinical encounter scenarios focused only on the primary care context. Our observations cannot be generalized to other medical contexts where the degree of EHR implementation and use could be different, and where patient expectations may vary and thus be associated with different patient preferences.

Patients included in our survey represent the patient population attending walk-in clinics in the French-speaking part of Switzerland and able to read and understand French. They were mostly younger than 50 years old, educated, and familiar with the use of computers and electronic devices. This could make our findings less generalizable as they do not represent the majority of the patients attending different practice settings in terms of age and socio-economic level. In addition, we collected little information about patients’ social and economic status apart from nationality, level of education, and language. Therefore, we are not able to assess further the impact of social and economic status on patients’ preferences regarding EHR behavior. However, previous studies did not observe significant differences regarding patients’ satisfaction concerning physicians’ EHR use between patients of different ages and educational status or racial/ethnic group .^3,6–8^ Non-French-speaking patients were excluded from our study as the video material and survey were in French. This could limit the generalizability of our results to allophone patients. However, previous studies in English-speaking countries did not observed significant difference between English and non-English-speaking patients over patients’ satisfaction regarding physicians’ EHR use .^3^

We tested patients’ preferences in only two places in the same Swiss town. It is possible that patients’ preferences could vary in other regions or countries as the culture and perceptions about computers may differ from one context to another.

Twenty percent of the participants made a double choice and selected more than one video. This finding is more difficult to interpret. We did not ask patients to explain their choices, as we wished to capture their spontaneous preferences. The next step would be to explore patients’ justifications for their preferences and their reasons for their potential hesitations (double choice) through semi-structured interviews. This could enrich our understanding of how computer use influences physician-patient interactions.

## Conclusion

Despite these limitations, our findings appear relevant for a large part of physician-patient computer interactions that take place in real life. Although our observations need to be repeated in different health care contexts, our results confirm patients’ positive attitudes towards the use of EHR by physicians as well as towards the experts’ recommendations regarding the use of EHR during the consultation. Our findings underline the need for physicians to pay greater attention to their behavior while using the EHR in order to stay patient-centered. Such recommendations should be more consistently taught during medical training since they are now increasingly evidence-based. More research is needed to explore qualitatively the reasons of patients’ preferences regarding the EHR-related behaviors. In particular, the importance of verbal and non-verbal communication while physicians are using EHR deserves further exploration .^51,52^

## Supplementary Information

ESM 1 (DOCX 20 kb)

ESM 2 (DOCX 17 kb)

## Data Availability

The datasets used and/or analyzed during the current study are available from the corresponding author upon reasonable request.
