# Supplementary material for: How Do Patients Want Us to Use the Computer During Medical Encounters?—A Discrete Choice Experiment Study
Source: J Gen Intern Med. 2021 Apr 26;36(7):1875–82. doi: 10.1007/s11606-021-06753-1 (PMC8298679; doi:10.1007/s11606-021-06753-1)
Supplement: Supplementary file 1 — (DOCX 20 kb) [file 11606_2021_6753_MOESM1_ESM.docx]

**Appendices**

**Discrete choice experiment (DCE) online survey**

Please watch the videos as if you were the patient in the consultation. Then, select your preferred sequence (choose the corresponding color) regarding the doctor’s behavior (you can select two sequences if you have no preferences between two of them)

Video A1

| Mr. Albert (70 years old.) consults Dr. Müller in emergency for a severe back pain that is not relieved by the medication prescribed by his doctor one week ago | | |
| --- | --- | --- |
| *Please watch the three video sequences before indicating your preferred choice* | | |
| □ Yellow | □ Green | □ Blue |

Video A2

| Mrs Bonvin (30 years old) consults Dr Jeanneret in an emergency room for a right hand wound | | |
| --- | --- | --- |
| *Please watch the three video sequences before indicating your preferred choice* | | |
| □Yellow | □Green | □ Blue |

Video B

| Mrs. Comte (45 years old) consults Dr. Favre for the first time for a headache. | | |
| --- | --- | --- |
| *Please watch the three video sequences before indicating your preferred choice* | | |
| □ Yellow | □ Green | □ Blue |

Video C

| Mr. Durand (18 years old) consults Dr Aubry for the first time for a skin rash that appeared three days ago | | |
| --- | --- | --- |
| *Please watch the two video sequences before indicating your preferred choice* | | |
| □ Yellow |  | □ Blue |

Video D

| Mrs. Erard (80 years old) consults with Dr. Rochat who replaces Dr. Dupont, the patient's doctor during her absence, because of a stomach pain evolving for the last 5 days. | | |
| --- | --- | --- |
| *Please watch the three video sequences before indicating your preferred choice* | | |
| □ Yellow | □ Green | □ Blue |

**Socio-demographic characteristics and experiences of computer use :**

| 1. You are... | | □ a man | □ a woman |
| --- | --- | --- | --- |
| 2. How old are you ? | | _ _ _ _ _ years old | |
| 3. What is your marital status? | | □ married  □ singled  □ divorced/separated  □ widow | |
| 4. What is your nationality ? | | _________ | |
| 5. What is your current professional situation? | | □ employee  □ independent  □ student or in vocational training  □ unemployed  □ retired or in early retirement, annuitant  □ housewife or househusband  □ other : _____________________ | |
| 6. What is the highest level of training you have completed? | | □ compulsory school  □ apprenticeship, vocational school  □ high school or other school of general culture  □ other higher vocational education and training (master's degree, federal certificate, higher vocational school, ETS, etc.)  □ university, university of applied sciences | |
| 7. What language do you feel most comfortable in? | | □ French  □ Spanish  □ English  □ Other | |
| 8. How many years have you been seeing your doctor/attending the division of primary care? | | ______years | |
| 9. How many times have you seen your doctor in the last 12 months? | | □ >12  □ 5-12  □ 3-4  □ 2  □ 1 | |
| 10. Does your doctor use a computer during the consultation? | | □ yes □ no | |
| 11. How do you rate your health? | | □ excellent  □ very good  □ good  □ average  □ poor | |
| 12. How familiar are you with computers, smartphones and tablets? | | □ very comfortable  □ comfortable  □ moderately comfortable  □ not very comfortable  □ not comfortable | |
| 13. How often do you use the following computer media? | | | |
| - computer | □ >1x/day □ >1x/week □ >1x/month □ >1x/6 months □ never | | |
| - internet | □ >1x/day □ >1x/week □ >1x/month □ >1x/6 months □ never | | |
| - email | □ >1x/day □ >1x/week □ >1x/month □ >1x/6 months □ never | | |
| - social networks (facebook, twitter, instagram…) | □ >1x/day □ >1x/week □ >1x/month □ >1x/6 months □ never | | |
| 14. What is your opinion regarding the use of computers by doctors? | | □ excellent thing  □ very good thing  □ good thing  □ no impact/no opinion  □ bad thing | |

Thank you very much for your participation!
