# Supplementary material for: How Do Patients Want Us to Use the Computer During Medical Encounters?—A Discrete Choice Experiment Study
Source: J Gen Intern Med. 2021 Apr 26;36(7):1875–82. doi: 10.1007/s11606-021-06753-1 (PMC8298679; doi:10.1007/s11606-021-06753-1)
Supplement: Supplementary file 2 — (DOCX 17 kb) [file 11606_2021_6753_MOESM2_ESM.docx]

**Supplementary Table: Results of the multinomial logistic regression of factors associated with patients’ choices regarding EHR-related behaviors. Data are relative risk ratios (p-values)**

| **Patient characteristics** | Age group ^*^ | | Male | 1^st^ language French † | Level of education ‡ | | ≥ 5 consult/yr | Computer use ≥ 1 per week | Own GP uses computer | Patient in favor of GP using computers | Patient familiar with e-devices | Study location: hospital |
| --- | --- | --- | --- | --- | --- | --- | --- | --- | --- | --- | --- | --- |
| **Behavior and variations** | 30-49 | >50 |  |  | Secondary | Tertiary |  |  |  |  |  |  |
| Typing - Biomedical content (video A1) |  |  |  |  |  |  |  |  |  |  |  |  |
| - Handwriting | baseline | baseline | baseline | baseline | baseline | baseline | baseline | baseline | baseline | baseline | baseline | baseline |
| - Intermittent typing | 1.55 (0.37) | 0.65 (0.46) | 0.46 (0.07) | 1.80 (0.24) | 0.80 (0.75) | 1.09 (0.90) | 2.13 (0.23) | 0.66 (0.55) | 0.94 (0.89) | 3.14 (0.02) | 0.84 (0.77) | 1.54 (0.32) |
| - Continuous typing | 2.02 (0.32) | 3.21 (0.11) | 0.64 (0.43) | 2.69 (0.16) | 0.46 (0.34) | 0.35 (0.21) | 0.56 (0.55) | 0.20 (0.04) | 2.65 (0.16) | 9.45 (0.01) | 4.36 (0.08) | 4.36 (0.28) |
| - Double choice | 0.64 (0.48) | 1.13 (0.84) | 0.37 (0.05)^§^ | 1.65 (0.38) | 0.76 (0.71) | 0.99 (0.99) | 1.29 (0.72) | 0.35 (0.14) | 1.41 (0.51) | 4.09 (0.02)^‖^ | 1.30 (0.70) | 1.98 (0.75) |
|  |  |  |  |  |  |  |  |  |  |  |  |  |
| Typing - Psychosocial content (video A2) |  |  |  |  |  |  |  |  |  |  |  |  |
| - Handwriting | baseline | baseline | baseline | baseline | baseline | baseline | baseline | baseline | baseline | baseline | baseline | baseline |
| - Intermittent typing | 0.44 (0.13) | 0.31 (0.04)^¶^ | 0.36 (0.02)^¶^ | 1.44 (0.47) | 0.96 (0.95) | 0.73 (0.60) | 0.84 (0.76) | 1.84 (0.29) | 1.20 (0.70) | 1.89 (0.28) | 0.71 (0.56) | 1.24 (0.61) |
| - Continuous typing | 0.67 (0.53) | 0.40 (0.19) | 0.78 (0.65) | 1.14 (0.81) | 1.06 (0.93) | 0.80 (0.76) | 1.46 (0.54) | 1.31 (0.69) | 0.74 (0.57) | 1.05 (0.93) | 1.04 (0.96) | 1.16 (0.83) |
| - Double choice | 1.44 (0.58) | 096 (0.96) | 0.48 (0.18) | 0.86 (0.80) | 4.25 (0.08) | 1.5 (0.64) | 0.61 (0.50) | 1.60 (0.54) | 2.00 (0.60) | 9.46 (0.06) | 2.34 (0.30) | 1.50 (0.45) |
|  |  |  |  |  |  |  |  |  |  |  |  |  |
| Contact (video B) |  |  |  |  |  |  |  |  |  |  |  |  |
| - Visual contact only | baseline | baseline | baseline | baseline | baseline | baseline | baseline | baseline | baseline | baseline | baseline | baseline |
| - Verbal contact only | 0.94 (0.92) | 1.15 (0.85) | 2.05 (0.22) | 0.79 (0.71) | 0.38 (0.21) | 0.60 (0.56) | 0.91 (0.90) | 0.77 (0.76) | 0.85 (0.80) | 1.56 (0.56) | 1.89 (0.37) | 0.30 (0.06) |
| - Visual + verbal contact | 2.25 (0.24) | 3.40 (0.11) | 1.40 (0.56) | 0.98 (0.98) | 0.46 (0.31) | 1.04 (0.97) | 0.38 (0.20) | 0.32 (0.16) | 1.04 (0.95) | 1.23 (0.79) | 2.92 (0.13) | 0.19 (0.01)^#^ |
| - Double choice | 1.23 (0.79) | 1.52 (0.61) | 1.88 (0.32) | 0.69 (0.60) | 0.32 (0.19) | 0.96 (0.97) | 0.64 (0.59) | 0.49 (0.44) | 0.36 (0.14) | 3.79 (0.14) | 3.07 (0.17) | 0.17 (0.01)^#^ |
|  |  |  |  |  |  |  |  |  |  |  |  |  |
| Signposting (video C) |  |  |  |  |  |  |  |  |  |  |  |  |
| - No signposting | baseline | baseline | baseline | baseline | baseline | baseline | baseline | baseline | baseline | baseline | baseline | baseline |
| - Signposting | 0.95 (0.85) | 0.79 (0.47) | 0.90 (0.67) | 0.83 (0.52) | 0.87 (0.67) | 1.26 (0.52) | 0.77 (0.43) | 1.21 (0.56) | 0.75 (0.28) | 1.02 (0.96) | 0.75 (0.41) | 0.67 (0.10) |
| - Double choice | 1.85 (0.36) | 1.69 (0.46) | 1.18 (0.74) | 0.88 (0.83) | 0.67 (0.63) | 1.56 (0.58) | 2.03 (0.22) | 4.12 (0.12) | 0.64 (0.40) | 1.08 (0.91) | 0.38 (0.14) | 0.55 (0.23) |
|  |  |  |  |  |  |  |  |  |  |  |  |  |
| Body position (video D) |  |  |  |  |  |  |  |  |  |  |  |  |
| - Bust facing patient / hands off the keyboard | baseline | baseline | baseline | baseline | baseline | baseline | baseline | baseline | baseline | baseline | baseline | baseline |
| - Bust facing patient / hands on the keyboard | 2.00 (0.22) | 0.87 (0.85) | 3.60 (0.01) | 3.22 (0.10) | 3.02 (0.18) | 3.79 (0.11) | 1.23 (0.78) | 0.07 (0.001)^**^ | 1.56 (0.40) | 3.90 (0.06) | 1.59 (0.56) | 0.62 (0.35) |
| - Bust facing computer / hands on the keyboard | 0.69 (0.59) | 1.09 (0.89) | 2.95 (0.05) | 049 (0.26) | 1.18 (0.84) | 1.57 (0.60) | 1.35 (0.71) | 0.24 (0.09) | 0.86 (0.79) | 1.52 (0.54) | 1.86 (0.43) | 0.47 (0.18) |
| - Double choice | 0.26 (0.05) | 0.69 (0.56) | 1.43 (0.47) | 0.78 (0.71) | 1.81 (0.46) | 3.08 (0.16) | 1.95 (0.35) | 0.19 (0.04) | 1.68 (0.33) | 3.16 (0.10) | 1.24 (0.77) | 1.31 (0.58) |

Comparison category: * < 30 years ; † all other languages; ‡ obligatory school

Interpretation: § Males less likely to hesitate between the two choices

‖ The relative risk of preferring intermittent or continuous typing over handwriting is significantly increased in those who are in favor of GPs using computers, in a consultation with biomedical content

¶ Older male patients less likely to favor intermittent typing

# Lower relative risk of choosing these two options could reflect different RA’s way of encouraging patients to make a choice

** Very low relative risk ratio of patient choosing this option (or to a lesser extent the double choice)if uses the computer frequently
